# 

**Published:** 1886-09

**Authors:** 


*Entered at the Post Office, at Austin, Texas, as Second class Matter.*

VOL. II.] **SEPTEMBER, 1886.** [NO. 3.

### DANIEL'S MEDICAL JOURNAL

SIT LUX

*Subscription: $2.00 a Year in Advance; - - Single Copies, 25 Cents.*

F. E. DANIEL, M. D., Editor and Publisher, AUSTIN, TEXAS.

NORTHERN OFFICE, 1217 FILBERT STREET, PHILADELPHIA.

